# Evolution beyond DNA: epigenetic drivers for evolutionary change?

**DOI:** 10.1186/s12915-023-01770-4

**Published:** 2023-12-29

**Authors:** Peter Sarkies

**Affiliations:** Department of Biochemistry, University of Oxford, South Parks Road, Oxford, OX13QU UK

## Abstract

**Epigenetics** **has been a buzzword in science for many years, but 20 years ago, the idea that epigenetic gene regulation could be a driver in evolutionary processes was only a mischievous glint in a Lamarckian eye. Now, however, theoretical and experimental studies have illuminated the possible roles that epigenetic information could play in evolution and we are on the cusp of testing this concept in ecologically relevant settings.**

## Epigenetics and evolution

What comes to mind when you think of epigenetics? Histone modifications? DNA methylation? Non-coding RNAs? Marbles and rubber sheets? All of these are familiar in textbook examples of epigenetics. Linking epigenetics and evolution, however, may not be so obvious. Nevertheless, advances over the last few years have provided some intriguing hints that epigenetic changes, potentially without DNA sequence changes, could directly drive evolutionary processes.

By conventional definition, an epigenetic change is one that alters gene expression states, without changing the DNA sequence [1]. Importantly, epigenetic changes are defined as being heritable through cell division, thus, mechanisms must exist that “copy” the epigenetic marks from a cell to its daughter [1]. This is a tough requirement because the epigenetic state must somehow survive the tumultuous rearrangements of the protein-DNA complex known as chromatin that takes place when DNA is replicated [2]. Not all changes that affect gene expression are heritable; however, molecular mechanisms have been described whereby some post-translational modifications of histone proteins and DNA methylation marks can be directly “copied”, analogously to the mechanism of DNA replication [2]. Moreover, in some organisms, short non-coding RNAs of 18–30 nucleotides that regulate gene expression can act in an epigenetic fashion because they recruit an enzyme called RNA-dependent RNA polymerase that can copy the RNAs to sustain their levels over multiple rounds of cell division [3].

Classically, the role of epigenetic inheritance is to allow cells to stably differentiate, whilst retaining the same genome. During development, cells diverge to take up different functions by turning on some genes and silencing others, and they remember these states for many subsequent divisions through epigenetic inheritance mechanisms. However, some epigenetic changes can also be transferred across generations, which is known as transgenerational epigenetic inheritance [1]. This is very well described in the model nematode worm *C. elegans*, where GFP or RFP transgenes introduced into the genome can be turned off epigenetically with either small RNAs or changes in histone modifications and the silenced state is maintained, sometimes for hundreds of generations totally independently of the original silencing [3].

Such dramatic examples of long-term silencing mediated solely by epigenetics lead to the question of whether some changes that contribute to evolutionary processes, adapting organisms to changing environments or even leading to formation of new species, might similarly be driven by epigenetics [4]. Testing this idea, though, is very difficult. The key problem is that evolution takes many generations during which many changes, both genetic and epigenetic, are expected. It’s often difficult to resolve what is the “cause”, even for genetic differences, and epigenetic differences are even harder to resolve as they might plausibly be secondary to differences in the genomic sequence rather than acting alone. We therefore have to study epigenetic differences as they arise in the laboratory, starting with genetically identical individuals.

The first key question to answer is whether epigenetic changes can last long enough in real populations to contribute to long-term epigenetic processes. Enormous strides towards answering this question were made in landmark experiments using plants [5, 6]. Key to these studies was an experimental design that allowed experimenters to reduce the size of the population that was transferred every generation down to just one individual — this is possible in the model plant Arabidopsis because it can self-fertilise. This means that natural selection, which usually weeds out deleterious changes in organisms due to “survival of the fittest” doesn’t work efficiently so that all changes, whether beneficial, neutral or deleterious, are equally likely to propagate so that an unbiased view of epigenetic changes could be obtained. These studies characterised changes in DNA methylation and found that these were indeed epigenetically inherited and differences accrued between different lineages. However, in a striking contrast to DNA sequence changes, the epigenetic differences were unstable, lasting only ~ 5 generations on average, in contrast to DNA sequence changes which are potentially irreversible.

Inspired by these pioneering plant studies my laboratory performed similar studies in animals, using the nematode worm *C. elegans* to investigate changes in small non-coding RNAs and changes in the structure of protein-DNA complexes (chromatin) [7]. Our results were similar to the plant studies: many epigenetic changes occurred and were heritable, but had a limited duration of only a few generations. Provocatively we found some biases in which genes were liable to accumulate epigenetic changes. Genes that were involved in the response to the environment, particularly genes that defend against toxic compounds in the environment, were particularly prone to acquire epigenetic change [7].

The fact that epigenetic changes occur in populations but have limited stability leads naturally to the question of whether they could contribute to real evolutionary processes driven by natural selection. It’s harder to imagine this than for a DNA sequence change because they are unstable so not all offspring will inherit the epigenetic change. However, theoretical studies have shown that, if there is a very strong selection for a particular phenotype, even an unstable epigenetic change could still drive evolution of the phenotype in the population [8]. Testing this concept in the lab, though, is still at an early stage.

The clearest experimental demonstration of epigenetic drivers of evolutionary processes comes from studies using yeast. Here, resistance to a drug (caffeine, which usually kills yeast) could be evolved solely through epigenetic changes [9]. This resistance was characteristically somewhat unstable in that it disappeared soon after the caffeine was removed. The cause of the resistance was an epigenetic difference which led to silencing of a particular gene and loss of this gene led to increased resistance to the drug. This could be shown to be completely independent of DNA sequence alterations. Yeast, as a single-celled organism, could be argued to be quite different from an animal or a plant where sexual reproduction and a long lifespan would raise the barrier for epigenetic transmission. However, intriguing work using worms has shown that epigenetic changes in small RNAs can drive some evolutionary processes by affecting mate preference in the laboratory [10], which acts as a tantalizing hint that evolution might be driven by epigenetics in multicellular organisms too.

What is needed now, though, is an effort to study epigenetics in a laboratory evolution experiment using a stimulus that is ecologically relevant, such as a natural toxin or a pathogen. By following epigenetic states in each generation at the same time as following the adaptation to the stimulus in multiple lineages independently, it will be possible to determine rigorously whether epigenetic changes are capable of driving the adaptation. Crucially, epigenetic changes can now be engineered in the lab, so whether any epigenetic change that occurs in the laboratory evolution was causative could be tested directly. These studies would potentially revolutionize our understanding of evolutionary processes, providing a richer view of how the differences in phenotype that drive natural selection arise and are maintained. The next 5–10 years will see a bumper crop of these experiments across multiple systems and I’m on tenterhooks!

## Data Availability

Not applicable.

## References

[1] Miska EA, Ferguson-Smith AC (2016). Transgenerational inheritance: Models and mechanisms of non-DNA sequence-based inheritance. Science.

[2] Alabert C, Jasencakova Z, Groth A (2017). Chromatin replication and histone dynamics. Adv Exp Med Biol.

[3] Cecere G (2021). Small RNAs in epigenetic inheritance: from mechanisms to trait transmission. FEBS Lett.

[4] Ashe A, Colot V, Oldroyd BP (1826). How does epigenetics influence the course of evolution?. Philos Trans R Soc Lond B Biol Sci..

[5] Schmitz RJ, Schultz MD, Lewsey MG, O’Malley RC, Urich MA, Libiger O (1979). Transgenerational epigenetic instability is a source of novel methylation variants. Science.

[6] Hagmann J, Becker C, Müller J, Stegle O, Meyer RC, Wang G (2015). Century-scale Methylome Stability in a Recently Diverged Arabidopsis thaliana Lineage. PLoS Genet..

[7] Wilson R, Le Bourgeois M, Perez M, Sarkies P (2023). Fluctuations in chromatin state at regulatory loci occur spontaneously under relaxed selection and are associated with epigenetically inherited variation in C. elegans gene expression. PLoS Genet..

[8] Charlesworth B, Jain K (2014). Purifying selection, drift, and reversible mutation with arbitrarily high mutation rates. Genetics.

[9] Torres-Garcia S, Yaseen I, Shukla M, Audergon PNCB, White SA, Pidoux AL (2020). Epigenetic gene silencing by heterochromatin primes fungal resistance. Nature.

[10] Toker IA, Lev I, Mor Y, Gurevich Y, Fisher D, Houri-Zeevi L (2022). Transgenerational inheritance of sexual attractiveness via small RNAs enhances evolvability in C. elegans. Dev Cell.

